# Three-year follow-up study reveals improved survival rate in NSCLC patients underwent guideline-concordant diagnosis and treatment

**DOI:** 10.3389/fonc.2024.1382197

**Published:** 2024-05-28

**Authors:** Huijuan Mu, Xing Yang, Yanxia Li, Bingzheng Zhou, Li Liu, Minmin Zhang, Qihao Wang, Qian Chen, Lingjun Yan, Wei Sun, Guowei Pan

**Affiliations:** ^1^ Institute of Preventive Medicine, China Medical University, Shenyang, China; ^2^ Institute of Chronic Diseases, Liaoning Provincial Center for Disease Control and Prevention, Shenyang, China; ^3^ Research Center for Universal Health, School of Public Health, China Medical University, Shenyang, China; ^4^ Department of Orthopaedic Surgery and Sports Medicine, Shengjing Hospital of China Medical University, Shenyang, China

**Keywords:** lung cancer, first course, diagnosis and treatment, guideline-concordant, survival

## Abstract

**Background:**

No studies in China have assessed the guideline-concordance level of the first-course of non-small cell lung cancer (NSCLC) diagnosis and treatment and its relationship with survival. This study comprehensively assesses the current status of guideline-concordant diagnosis (GCD) and guideline-concordant treatment (GCT) of NSCLC in China and explores its impact on survival.

**Methods:**

First course diagnosis and treatment data for NSCLC patients in Liaoning, China in 2017 and 2018 (n=1828) were used and classified by whether they underwent GCD and GCT according to Chinese Society of Clinical Oncology (CSCO) guidelines. Pearson’s chi-squared test was used to determine unadjusted associations between categorical variables of interest. Logistic models were constructed to identify variables associated with GCD and GCT. Kaplan–Meier analysis and log-rank tests were used to estimate and compare 3-year survival rates. Multivariate Cox proportional risk models were constructed to assess the risk of cancer mortality associated with guideline-concordant diagnosis and treatment.

**Results:**

Of the 1828 patients we studied, 48.1% underwent GCD, and 70.1% underwent GCT. The proportions of patients who underwent both GCD and GCT, GCD alone, GCT alone and neither GCD nor GCT were 36.7%, 11.4%, 33.5% and 18.4%, respectively. Patients in advanced stage and non-oncology hospitals were significantly less likely to undergo GCD and GCT. Compared with those who underwent neither GCD nor GCT, patients who underwent both GCD and GCT, GCD alone and GCT alone had 35.2%, 26.7% and 35.7% higher 3-year survival rates; the adjusted lung cancer mortality risk significantly decreased by 29% (adjusted hazard ratio[aHR], 0.71; 95% CI, 0.53–0.95), 29% (aHR, 0.71; 95% CI, 0.50–1.00) and 32% (aHR, 0.68; 95% CI, 0.51–0.90).

**Conclusion:**

The 3-year risk of death is expected to be reduced by 29% if patients with NSCLC undergo both GCD and GCT. There is a need to establish an oncology diagnosis and treatment data management platform in China to monitor, evaluate, and promote the use of clinical practice guidelines in healthcare settings.

## 1 Introduction

Lung cancer is one of the most common malignant tumors. In 2020, there were approximately 2.207 million new lung cancer patients worldwide, ranking second in new malignant tumors, and 1.796 million new lung cancer deaths, ranking first among all malignant tumors (1). At the same time, the survival rate of lung cancer worldwide is low, with a 5-year survival rate of less than 30% (2). In addition to the biological factors that affect the prognosis of lung cancer (such as age, sex, disease stage, etc.) (3–5), the diagnosis and treatment of lung cancer is also critical (6–9).

During recent decades, authoritative clinical practice guidelines for the diagnosis and treatment of lung cancer, including those from the American College of Chest Physicians (ACCP) (10), American Society for Clinical Oncology (ASCO) (11), National Cancer Institute (NCI) (12), European Society for Medical Oncology (ESMO) (13), and other academic societies, have been adopted globally for lung cancer diagnosis and treatment. The clinical practice guidelines compile existing evidence and expert consensus and can be viewed as the established basis for guideline-concordant care (GCC) for lung cancer (14). Studies of clinical practice patterns in the United States have documented differences in lung cancer management in terms of age, race, education, comorbidities, insurance and type of hospital (15–22). In particular, some studies based on Surveillance, Epidemiology and End Results (SEER) and the National Cancer Data-base (NCDB) have demonstrated significant improvements in survival outcomes for patients with lung cancer undergoing GCC (23, 24).

Nadpara et al. reported that 44.7% of old lung cancer patients in the SEER-Medicare database (2002–2007) underwent GCC, and the three-year median survival time was significantly longer for patients undergoing GCC (747 days) than non-GCC patients (260 days) (23). A study specifically investigating the association between GCC and overall survival in patients with locally advanced non-small cell lung cancer (NSCLC) found that 23% of patients underwent GCC, that socioeconomic factors, including lack of insurance and geographic location, were associated with non-GCC, that patient- and disease-specific factors, including advanced adenocarcinoma histology and gender, were also associated with non-GCC, and that non-GCC patients had higher mortality rates than GCC patients (hazard ratio [HR], 1.42) (24). However, it is unclear whether these results are broadly generalizable given the limited comparability of existing studies, which typically examine only specific subgroups of clinical cases, define guideline-concordant lung cancer care based primarily on treatment undergone, and fail to capture the appropriateness of the lung cancer diagnostic process prior to undergoing treatment (23–25).

In China, the number of new cases of lung cancer in 2018 was 781,000 (85% of which were NSCLC), the highest in the world, but the level of survival is in the low, with a 3-year survival rate of only 19.8% (26, 27). Although many oncology diagnostic and treatment guidelines and related documents have been published in China in recent years, and the “Three-year Action Plan for Cancer Prevention and Treatment in China (2015–2017)” issued in 2016 emphasized the standardization of oncology diagnosis and treatment practices (28), there is no authoritative database similar to SEER containing information on the first course of diagnosis and treatment of tumors and survival, the level of guideline-concordant diagnosis and treatment of NSCLC has not been fully evaluated, and the associated health outcomes are unclear. Liaoning Province, located in northeastern China, is an important industrial area with a high incidence of lung cancer (29).

Therefore, in this study, we used data from patients with NSCLC in Liaoning Province with a similar structure to SEER, with the following study objectives:

1. To assess the level of guideline concordance, distribution characteristics and non-concordance issues for the first course of diagnosis and treatment;
2. To assess the impact of guideline-concordant diagnosis (GCD) and guideline-concordant treatment (GCT) on survival, including GCD alone, GCT alone and both GCD and GCT.

## 2 Methods

### 2.1 Data collection

**Figure 1:**
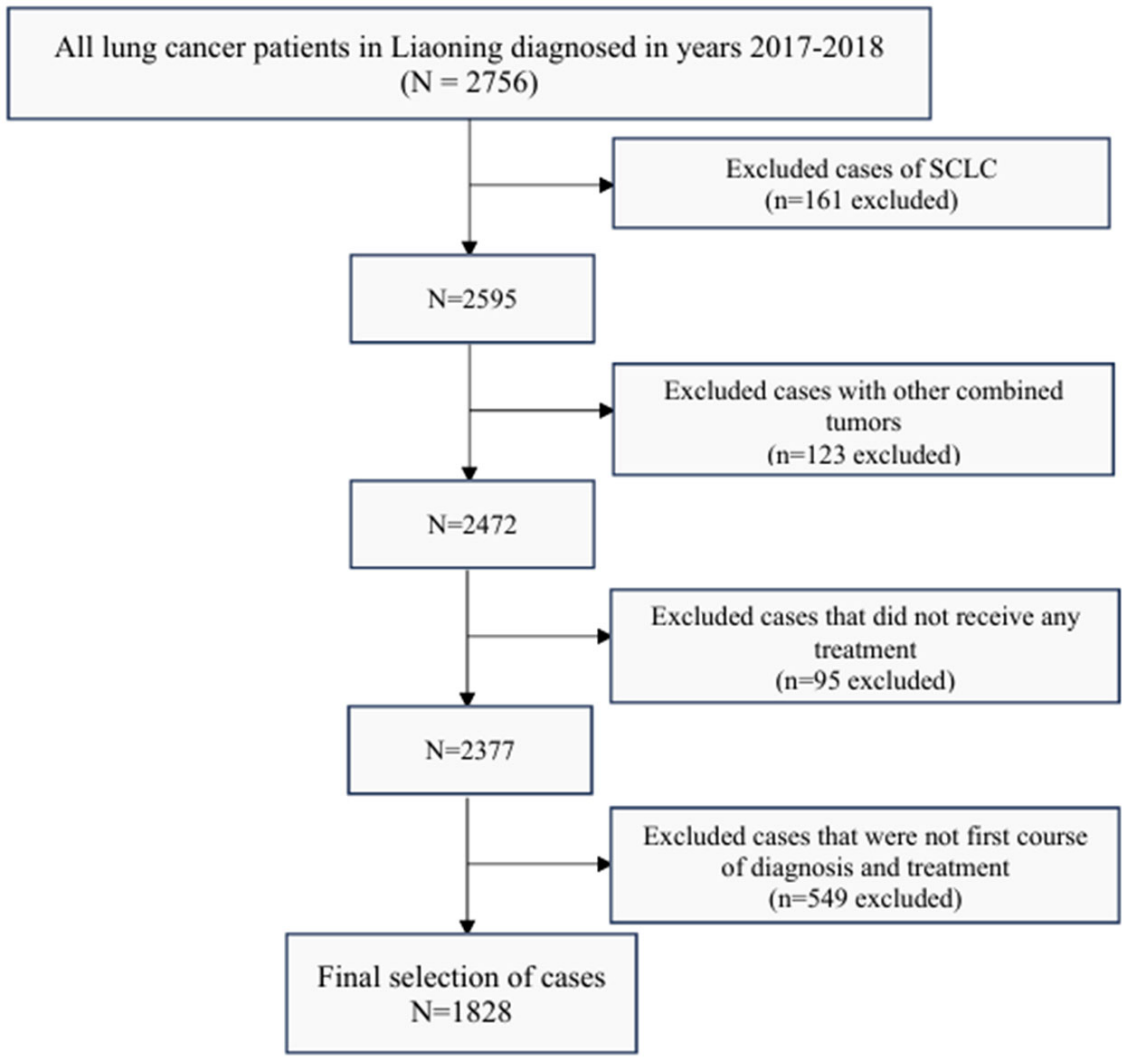
Case selection flowchart. NSCLC, non-small cell lung cancer.

This study was conducted using multi-stage cluster sampling to select 2756 patients with lung cancer from 20 hospitals in Liaoning Province who were diagnosed and treated with their first course between January 1, 2017 and December 31, 2018. We define the first course of diagnosis and treatment as the first diagnosis and cancer-oriented treatment administered within four months of diagnosis (30). Patients with non-small cell carcinoma by pathological type were selected; patients with other organ insufficiency, patients with other malignant tumors, and patients who gave up treatment were excluded. Based on the inclusion and exclusion criteria, we excluded 161 cases of SCLC, 123 cases with other combined tumors, 95 cases that did not receive any treatment, and 549 cases that were not the first course of diagnosis and treatment, resulting in the final selection of 1,828 patients with NSCLC (**Figure 1**).

First course diagnosis and treatment monitoring data: to ensure comparability with the general population in the United States, this study took SEER dataset monitoring and evaluation system as the main framework, combined with the latest and authoritative diagnosis and treatment guidelines for lung cancer in China, in which the monitoring and evaluation plan for lung cancer is formulated. This project invited experts in the diagnosis and treatment of lung cancer from surgery, internal medicine, pathology and other specialties to set up a lung cancer expert group to determine and evaluate the guideline consistency questionnaire for the first course of diagnosis and treatment. The questionnaire included 22 diagnostic indicators, 16 surgical indicators, 38 radiotherapy and chemotherapy indicators, 19 targeted therapy indicators and other indicators. It was completed by physicians and continuously validated for data quality and accuracy through internal monitoring, data quality reviews, and on-site surveys.

Survival outcome follow-up surveys: passive follow-up (Cancer Death Registration System of Liaoning Provincial Center for Disease Control and Prevention) and active follow-up (telephone follow-up) were combined to investigate the three-year survival outcomes of patients with lung cancer; the last follow-up time was December 31, 2021. Of the 1828 patients included in the analysis, 149 patients were lost due to moving out of the area, for a loss rate of 8.15% in this study.

### 2.2 Definition of guideline-concordant diagnosis and treatment

According to evidence-based medicine and in combination with the country’s national conditions, the Chinese Society of Clinical Oncology (CSCO) first issued the “Guidelines for the diagnosis and treatment of primary lung cancer” in 2016 (31). This study refers to the “Guidelines for the diagnosis and treatment of primary lung cancer” of the Chinese Society of Clinical Oncology (2016 edition) and the updated content of the 2017 edition, combined with expert opinions to define guideline-concordant diagnosis and treatment (**Table 1**).

**Table 1:** Description of GCD and GCT by each clinical subgroup.

Stage subgroup	GCD	GCT
I	appropriate clinical TNM staging + pathotyping	surgery (lobectomy) or SBRT
II		surgery + chemotherapy or radiotherapy ± chemotherapy
III		surgery + chemotherapy or radiotherapy + chemotherapy or surgery + radiotherapy + chemotherapy
IV	appropriate clinical TNM staging + pathotyping + molecular pathology testing	chemotherapy or targeted therapy or radiotherapy + chemotherapy

GCD, guideline-concordant diagnosis; GCT, guideline-concordant treatment; SBRT, stereotactic body radiotherapy.

GCD: defined as correct clinical TNM staging and pathotyping prior to treatment, and molecular pathology testing in advanced stages. Clinical staging is completed according to the 8th edition of the AJCC TNM staging system, T (primary tumor staging: T0, T1s, T1a-T4), N (regional lymph node staging: N0-N2), M (distant metastasis staging: Mx-M1c), and then divided into I A, I B, II A, II B, III A, III B, III C, IV A, IV B (32). To clarify the small-cell lung carcinoma (SCLC) and NSCLC, and to clarify the squamous and adenocarcinoma in NSCLC. After histologic diagnosis, sufficient tissue should be preserved for molecular testing, and treatment should be guided according to molecular typing.

GCT: after accurate staging by a panel of experts based on tumor size, lymph node metastasis and information on distant metastasis, the first course of treatment that patients should undergo at each stage was determined according to CSCO guidelines:

Stage I: Although recent studies have pointed out that sublobar resection is not inferior to lobectomy in terms of disease-free survival (33), the current clinical guidelines in China still recommend anatomic lobectomy as the main minimal treatment for stage I; Stereotactic Body Radiotherapy (SBRT) is an effective, low-division, non-invasive ablative treatment that delivers high doses of radiation only to specific targets and has a high rate of tumor control, well tolerated by normal tissues, and is the basic treatment strategy for patients who are not suitable for surgery (34, 35). Therefore, both lobectomy and SBRT are considered to be GCT for stage I NSCLC. SBRT is defined as chest radiation therapy with a total radiation dose of 45 grays or more delivered in fractions of 5 or less.
Stage II: the standard of care for stage II NSCLC is surgical resection combined with chemotherapy, if the patient is unable to tolerate surgery, the minimum recommendation is radiotherapy, possibly with adjuvant chemotherapy.
Stage III: stage III NSCLC is a very heterogeneous group of diseases and the minimum recommended treatment depends on operability, if surgery is possible, the minimum recommendation is surgery combined with chemotherapy; for the majority of patients who cannot undergo surgery, the minimum recommendation is a combination of radiotherapy and chemotherapy.
Stage IV: for driver gene-positive advanced NSCLC, the recommended treatment is targeted therapy, and for patients whose genotype cannot be specified for various reasons the minimum recommended treatment is conventional chemotherapy.

### 2.3 Statistical analysis

We used clinical stage at diagnosis for creating clinical subgroups, because pathological stage can only be known after the outcome of interest (initial treatment) has occurred. We assessed the proportion of GCD and GCT for each clinical subgroup, and then intersected diagnosis and treatment to assess the proportion of both GCD and GCT, GCD alone, GCT alone and neither GCD or GCT. Pearson’s chi-squared test was used to determine unadjusted associations between categorical variables of interest. The logistic regression model was used to predict GCD and GCT status, and propensity scores for sex, age, pathological type, stage, area, hospital type, etc. were calculated. Kaplan–Meier analysis and log-rank tests were used to estimate and compare 3-year survival rates. Hazard ratios (HRs) were calculated using Cox proportional hazard modeling. All analyses were performed using SPSS 25.0; the threshold for statistical significance for all tests was 0.05.

## 3 Results

### 3.1 Patient characteristics and disparities in undergoing guideline-concordant diagnosis and treatment

**Table 2** shows the distribution of clinical and sociodemographic characteristics of these patients. There are slightly more women than men (55.0% vs. 45.0%), and there are roughly equal numbers of patients younger than 60 and older than 60. The pathological type of most tumors was lung adenocarcinoma (LUAD) (77.9%), followed by lung squamous cell carcinoma (LUSC) (12.8%); large cell carcinoma, atypical carcinoma and adenosquamous carcinoma were combined with others (9.3%), and more than half of the patients were in stage I (63.7%). Most patients were from urban areas (62.0%), only 18.5% of patients presented at specialist oncology hospitals.

**Table 2:** Descriptive characteristics of lung cancer cases by GCD and GCT.

Characteristics		N (%)	Diagnosis			Treatment		
			GCD N (%)	Non-GCD N (%)	*P*-value	GCT N (%)	Non-GCT N (%)	*P*-value
Overall		1828 (100.0)	879 (48.1)	949 (51.9)		1282 (70.1)	546 (29.9)	
Sex	Male	822 (45.0)	413 (47.0)	409 (43.1)	0.095	541 (42.2)	281 (51.5)	**<0.001**
	Female	1006 (55.0)	466 (53.0)	540 (56.9)		741 (57.8)	265 (48.5)	
Age	≤60	887 (48.5)	423 (48.1)	464 (48.9)	0.743	637 (49.7)	250 (45.8)	0.127
	>60	941 (51.5)	456 (51.9)	485 (51.1)		645 (50.3)	296 (54.2)	
Pathological type	LUAD	142 (77.9)	739 (84.1)	685 (72.2)	**<0.001**	1024 (79.9)	400 (73.3)	**<0.001**
	LUSC	234 (12.8)	123 (14.0)	111 (11.7)		140 (10.9)	94 (17.2)	
	Others	170 (9.3)	17 (1.9)	153 (16.1)		118 (9.2)	52 (9.5)	
Stage	I	1165 (63.7)	627 (71.3)	538 (56.7)	**<0.001**	990 (77.2)	175 (32.1)	**<0.001**
	II	236 (12.9)	98 (11.1)	138 (14.5)		117 (9.1)	119 (21.8)	
	III	250 (13.7)	133 (15.1)	117 (12.3)		121 (9.4)	129 (23.6)	
	IV	177 (9.7)	21 (2.4)	156 (16.4)		54 (4.2)	123 (22.5)	
Area	Urban	1134 (62.0)	529 (60.2)	605 (63.8)	0.116	795 (62.0)	339 (62.1)	0.976
	Rural	694 (13.7)	350 (39.8)	344 (36.2)		487 (38.0)	207 (37.9)	
Oncology Specialist Hospital	Yes	337 (18.5)	308 (35.0)	29 (3.1)	**<0.001**	261 (20.4)	76 (13.9)	**<0.001**
	No	1491 (81.5)	571 (65.0)	920 (96.9)		1021 (79.6)	470 (86.1)	

LUAD, lung adenocarcinoma; LUSC, lung squamous cell carcinoma. Bold values indicate statistically significant values.

In the study population, less than half of the patients (48.1%) underwent GCD, but most patients (70.1%) underwent GCT. Pathological type, stage, city type, and hospital type were associated with undergoing GCD (p < 0.001); sex, pathology type, stage and hospital type were associated with undergoing GCT (p < 0.001). In addition, the proportions of patients who underwent both GCD and GCT, GCD alone, GCT alone, and neither GCD nor GCT were 36.7%, 11.4%, 33.5%, and 18.4%, respectively, and there were statistically significant differences for sex, pathology type, stage and hospital type. However, there was no difference in the distribution of patients who lost follow-up in the four groups (**Table 3**).

**Table 3:** Descriptive characteristics of lung cancer cases by both GCD and GCT, GCD alone, GCT alone, or neither.

Characteristics		Both N (%)	GCD alone N (%)	GCT alone N (%)	Neither N (%)	*P*-value
Overall		670 (36.7)	209 (11.4)	612 (33.5)	337 (18.4)	
Sex	Male	309 (46.1)	104 (49.8)	232 (37.9)	177 (52.5)	**<0.001**
	Female	361 (53.9)	105 (50.2)	380 (62.1)	160 (47.5)	
Age	≤60	327 (48.8)	96 (45.9)	310 (50.7)	154 (45.7)	0.428
	>60	343 (51.2)	113 (54.1)	302 (49.3)	183 (54.3)	
Pathological type	LUAD	570 (85.1)	169 (80.9)	454 (74.2)	231 (68.5)	**<0.001**
	LUSC	87 (13.0)	36 (17.2)	53 (8.7)	58 (17.2)	
	Others	13 (1.9)	4 (1.9)	105 (17.2)	48 (14.2)	
Stage	I	516 (77.0)	111 (53.1)	474 (77.5)	64 (19.0)	**<0.001**
	II	60 (9.0)	38 (18.2)	57 (9.3)	81 (24.0)	
	III	77 (11.5)	56 (26.8)	44 (7.2)	73 (21.7)	
	IV	17 (2.5)	4 (1.9)	37 (6.0)	119 (35.3)	
Area	Urban	399 (59.6)	130 (62.2)	396 (64.7)	209 (62.0)	0.307
	Rural	271 (40.4)	79 (37.8)	216 (35.3)	128 (38.0)	
Oncology Specialist Hospital	Yes	248 (37.0)	60 (28.7)	13 (2.1)	16 (4.7)	**<0.001**
	No	422 (63.0)	149 (71.3)	599 (97.9)	321 (95.3)	
Loss to Follow-up	Yes	51 (7.6)	17 (8.1)	46 (7.5)	35 (10.4)	0.417
	No	619 (92.4)	192 (91.9)	566 (92.5)	302 (89.6)	

Bold values indicate statistically significant values.

### 3.2 Factors associated with diagnosis and treatment patterns

The results of the unordered multicategorical logistic regression analysis showed that the factors influencing the diagnosis and treatment patterns were pathology type, stage, and hospital type. Taking “Neither” as a control, LUAD was associated with “Both” (adjusted odds ratio [aOR], 14.54; 95% CI, 6.79–31.12) and “GCD alone” (aOR, 16.29; 95% CI, 5.30–50.08), similarly, LUSC was associated with “Both” (aOR, 12.91; 95% CI, 5.55–30.02) and “GCD alone” (aOR, 13.58; 95% CI, 4.13–44.74). Stages I was associated with “Both” (aOR, 39.13; 95% CI, 10.35–68.42), “GCD alone” (aOR, 34.17; 95% CI, 18.72–97.05) and “GCT alone” (aOR, 22.86; 95% CI, 14.46–36.15), Stages II was associated with “Both” (aOR, 6.75; 95% CI, 3.27–13.92), “GCD alone” (aOR, 17.91; 95% CI, 5.88–54.58) and “GCT alone” (aOR, 2.24; 95% CI, 1.36–3.72), Stages III was associated with “Both” (aOR, 10.04; 95% CI, 4.90–20.58), “GCD alone” (aOR, 30.60; 95% CI, 10.14–92.32) and “GCT alone” (aOR, 1.99; 95% CI, 1.16–3.39). In addition, oncology specialist hospital was associated with “Both” (aOR, 26.95; 95% CI, 14.20–51.57) and “GCD alone” (aOR, 15.84; 95% CI, 8.00–31.35) (**Table 4**).

**Table 4:** Multifactorial logistic regression of factors influencing acceptance of both GCD and GCT, GCD alone, and GCT alone (compare with Neither).

Characteristics	Both aOR (95%CI)	GCD alone aOR (95%CI)	GCT alone aOR (95%CI)
Sex (ref = Female)			
Male	1.21 (0.85–1.72)	1.10 (0.72–1.66)	0.888 (0.63–1.23)
Age (ref = >60)			
≤60	0.88 (0.64–1.22)	0.81 (0.55–1.19)	0.98 (0.72–1.34)
Pathological type (ref = Others)			
LUAD	14.54 (6.79–31.12)^***^	16.29 (5.30–50.08)^***^	0.88 (0.56–1.39)
LUSC	12.91 (5.55–30.02)^***^	13.58 (4.13–44.74)^***^	0.81 (0.45–1.47)
Stage (ref =IV)			
I	39.13 (10.35–68.42)^***^	34.17 (18.72–97.05)^***^	22.86 (14.46–36.15)^***^
II	6.75 (3.27–13.92)^***^	17.91 (5.88–54.58)^***^	2.24 (1.36–3.72)^***^
III	10.04 (4.90–20.58)^***^	30.60 (10.14–92.32)^***^	1.99 (1.16–3.39)^***^
Area (ref = Rural)			
Urban	0.86 (0.62–1.20)	0.98 (0.66–1.45)	1.03 (0.75–1.41)
Oncology Specialist Hospital (ref = No)			
yes	26.95 (14.20–51.17)^***^	15.84 (8.00–31.35)^***^	0.78 (0.35–1.72)

aOR, adjusted odds ratio.

^***^P < 0.001.

### 3.3 Non-guideline concordant diagnosis and treatment patterns

As shown in **Table 5**, inappropriate staging was the most common non-GCD in stages I–III, accounting for 44.1%, 56.4% and 40.4%, while in stage IV, lack of molecular pathological testing was the most common non-GCD (79.0%). Sublobectomy was the most common non-GCT in stage I, accounting for 96.6%, and the most common non-GCT in stage II and III was surgery only, accounting for 95.8% and 60.5%; however, in advanced NSCLC, non-targeted therapy with unknown driver genes was the most common non-GCT, accounting for 69.1%.

**Table 5:** Non-guideline concordant diagnosis and treatment patterns by stage.

Stage groups	Non-GCD N (%)		Non-GCT N (%)	
I	inappropriate staging ^a^	513 (95.4)	surgery (sublobectomy)	169 (96.6)
	inappropriate pathotyping ^b^	25 (4.6)	chemotherapy only	6 (3.4)
II	inappropriate staging	133 (96.4)	surgery only	114 (95.8)
	inappropriate pathotyping	5 (3.6)	chemotherapy only	5 (4.2)
III	inappropriate staging	100 (85.5)	surgery only	78 (60.5)
			radiotherapy only	14 (10.9)
	inappropriate pathotyping	17 (14.5)	chemotherapy only	17 (13.2)
			surgery + radiotherapy	20 (15.4)
IV	inappropriate staging	4 (2.6)	surgery only	17 (13.8)
	inappropriate pathotyping	12 (7.7)	radiotherapy only	21 (17.1)
	lake of molecular pathology testing	140 (89.7)	non-targeted therapy	85 (69.1)

a “inappropriate staging” included incorrect noting of TNM staging in the medical records, incomplete TNM staging (lack of T/N/M), and no staging done at all.

b “inappropriate pathotyping” included incorrect or no noting of pathotyping in the medical record.

### 3.4 Survival associated with undergoing guideline-concordant diagnosis and treatment

**Figure 2:**
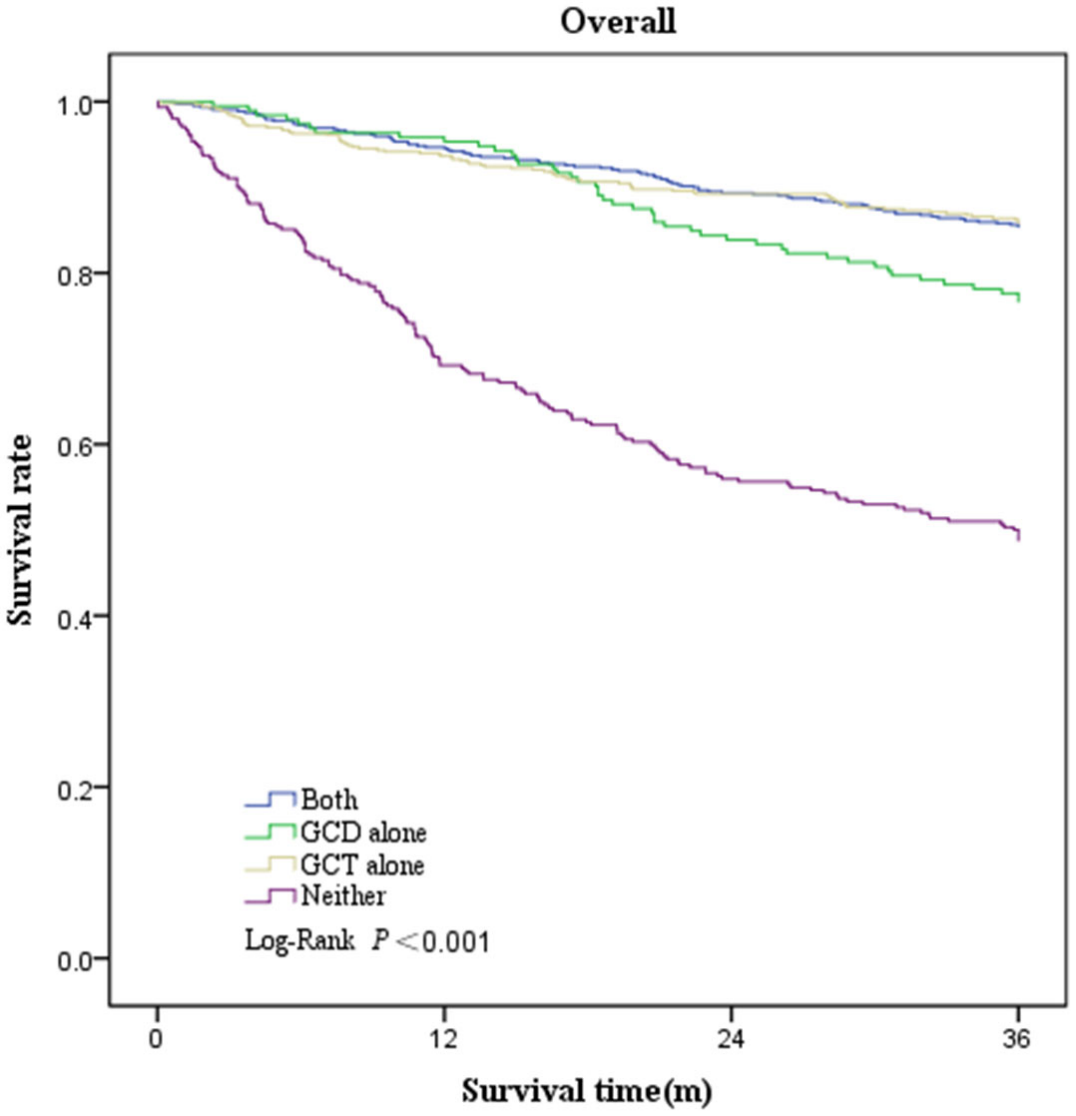
Kaplan–Meier plot showing overall survival rate among patients underwent both GCD and GCT, GCD alone, GCT alone, or Neither.

**Figure 3:**
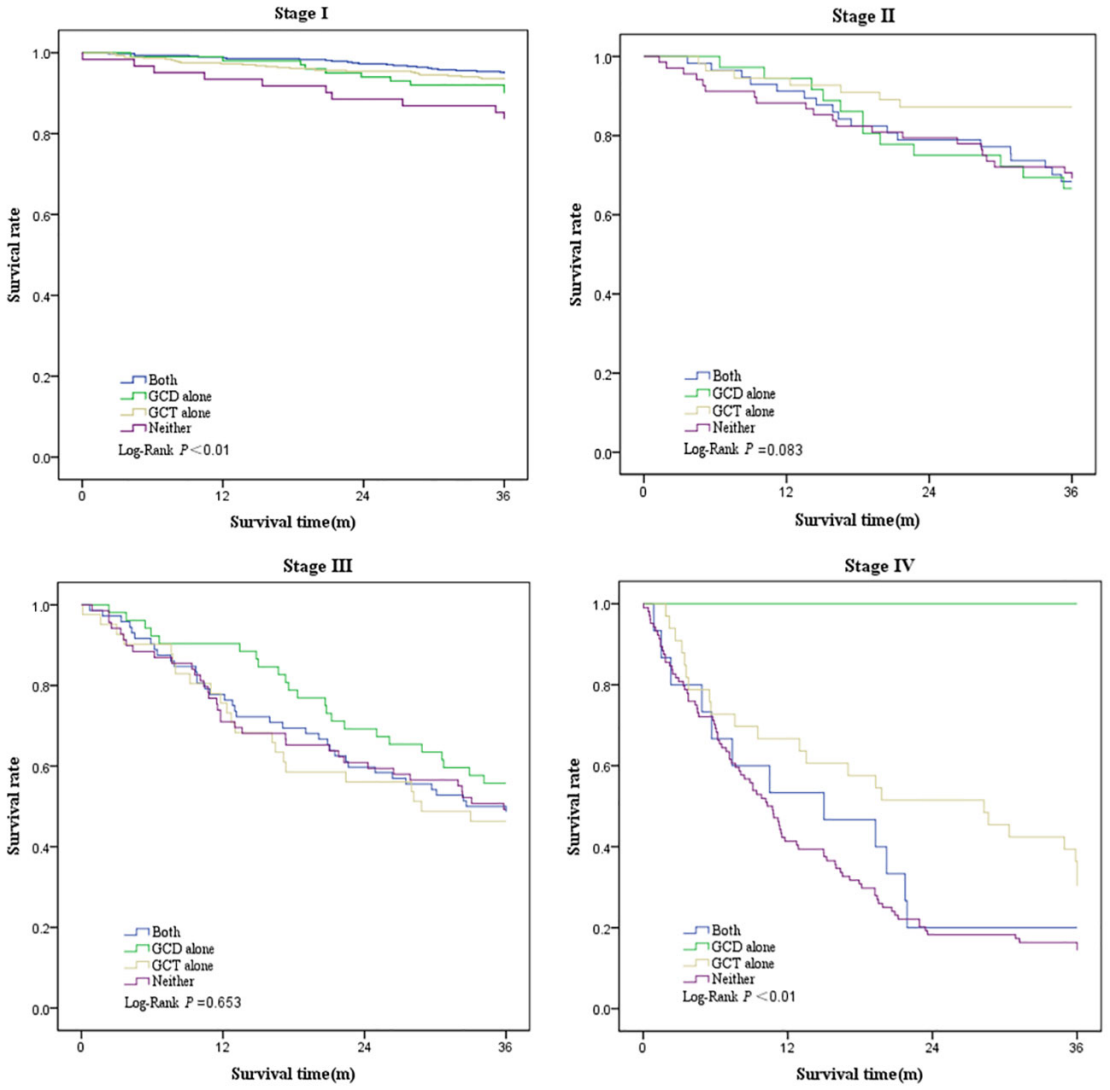
Kaplan–Meier plot showing survival rate by clinical stage among patients underwent both GCD and GCT, GCD alone, GCT alone, or Neither.

Survival was greatest in patients who underwent GCT alone (3-year overall survival, 79.2%; 95% CI, 76.0%–82.5%), followed by patients who underwent both GCD and GCT (3-year overall survival, 78.8%; 95% CI, 75.7%–81.9%), and patients who underwent GCD alone (3-year overall survival, 70.3%; 95% CI, 64.1%–76.6%), and worst in those who underwent neither (3-year overall survival, 43.6%; 95% CI, 38.3%–48.9%) (**Table 6**, **Figure 2**). Three-year survival analysis for each clinical stage showed that for stage I patients, survival was significantly higher for those who underwent both GCD and GCT than for the other patterns of care(3-year survival, 87.4%; 95% CI, 84.5%–90.3%), but this difference in survival was not significant in other stages of NSCLC patients (**Table 6**, **Figure 3**).

**Table 6:** 3-year survival rate of patients underwent both GCD and GCT, GCD alone, GCT alone, or Neither.

	Both	GCD alone	GCT alone	Neither	*P*-value
Overall	78.8% (75.7%–81.9%)	70.3% (64.1%–76.6%)	79.2% (76.0%–82.5%)	43.6% (38.3%–48.9%)	**<0.001**
Stage I	87.4% (84.5%–90.3%)	81.1% (73.8%–88.4%)	86.1% (83.0%–89.2%)	79.7% (69.8%–89.5%)	**0.004**
Stage II	65.0% (52.9%–77.1%)	63.2% (47.8%–78.5%)	84.2% (74.7%–93.7%)	58.0% (47.3%–68.8%)	0.083
Stage III	45.5% (34.3%–56.6%)	51.8% (38.7%–64.9%)	43.2% (28.5%–57.8%)	46.6% (35.1%–58.0%)	0.653
Stage IV	17.6% (0.5%–35.8%)	100.0% (–)	27.0% (12.7%–41.3%)	12.6% (6.6%–18.6%)	**0.004**

Bold values indicate statistically significant values.

Of the overall patients, after adjusting for age, sex, pathological type, and stage, patients undergoing both GCD and GCT showed a 29% reduction in the risk of death compared with those undergoing neither (aHR, 0.71; 95% CI, 0.53–0.95), patients who underwent GCD alone showed a lower hazard of death compared with those who underwent neither (aHR, 0.71; 95% CI, 0.50–1.00), and those who received only GCT showed significantly better survival than those receiving neither (aHR, 0.68; 95% CI, 0.51–0.90).The results of the subgroup analysis showed that the risk of death was significantly lower in patients in stage I who underwent both GCD and GCT or GCT alone than in those who underwent neither (both vs. neither: aHR, 0.28 [95% CI, 0.13–0.59]; GCT alone vs. neither: aHR, 0.37 [95% CI, 0.18–0.75]). In stages II and IV, the risk of death was significantly lower in patients who underwent GCT (both vs. neither: aHR, 0.39 [95% CI, 0.17–0.92]; both vs. neither: aHR, 0.60 [95% CI, 0.37–0.96]) (**Table 7**).

**Table 7:** Results of Cox proportional hazard models.

Characteristics	Overall aHR (95%CI)	Stage I aHR (95%CI)	Stage II aHR (95%CI)	Stage III aHR (95%CI)	Stage IV aHR (95%CI)
Pattern (ref = Neither)					
Both	0.71 (0.53–0.95)^*^	0.28 (0.13–0.59)^***^	0.93 (0.48–1.79)	0.93 (0.58–1.49)	0.88 (0.48–1.61)
GCD alone	0.71 (0.50–1.00)^*^	0.56 (0.23–1.37)	0.93 (0.44–1.93)	0.77 (0.45–1.31)	0.00 (–)
GCT alone	0.68 (0.51–0.90)^**^	0.37 (0.18–0.75)^**^	0.39 (0.17–0.92)^*^	1.21 (0.70–2.07)	0.60 (0.37–0.96)^*^
Sex (ref = female)					
male	1.60 (1.26–2.02)^***^	2.00 (1.20–3.33)^**^	1.66 (0.91–3.03)	1.60 (1.00–2.55)^*^	1.30 (0.88–1.93)
Age (ref = >60)					
≤60	0.74 (0.60–0.92)^***^	0.66 (0.41–1.07)	0.86 (0.50–1.47)	0.77 (0.53–1.12)	0.71 (0.48–1.04)
Pathological type (ref = Others)					
LUAD	0.74 (0.53–1.04)	0.50 (0.24–1.04)	0.49 (0.24–1.00)^*^	1.40 (0.66–2.99)	0.78 (0.44–1.40)
LUSC	1.21 (0.83–1.77)	2.20 (0.97–4.96)	0.62 (0.27–1.42)	1.59 (0.74–3.44)	1.17 (0.57–2.38)
Stage (ref = IV)					
I	0.05 (0.04–0.07)^***^				
II	0.18 (0.13–0.25)^***^				
III	0.36 (0.28–0.48)^***^				

aHR, adjusted hazard ratio.

^*^P < 0.05.^**^P < 0.01.^***^P < 0.001.

## 4 Discussion

There are major differences between China and Europe and the United States in terms of health insurance, new drugs and individual FDA-approved therapies, as well as varying levels of economic and medical resources across China (36–38). CSCO guidelines not only take into account the imbalance of development in various regions of China, but also make relevant adjustments to the guidelines in many aspects such as the accessibility of drugs and treatment measures and the value of tumor treatment, and therefore can be said to be the most suitable guidelines for China’s national conditions (31). To the best of our knowledge, this study is the first in China to assess the guideline concordance in lung cancer diagnosis and treatment using first course lung cancer diagnosis and treatment data with a similar structure to SEER data and in combination with Chinese clinical guidelines, the results are more comprehensive and credible than previous case report studies in China.

Diagnosis is a prerequisite for treatment, but there is now no international common standard for assessing guideline concordant diagnosis. In this study, three evaluation components were identified according to the guidelines: clinical TNM staging, pathological staging, and molecular pathology examination in advanced stages. Accurate clinical TNM staging and pathology reporting are important for clinical treatment options and disease outcomes (39, 40), but from our results, more than half of patients did not undergo a GCD, the majority of these were attributed to inappropriate staging, accounting for 41.0% of all patients, a decrease from the 63.1% reported in the 1992–1999 case review in Shanghai, China (41), reflecting the importance of quality of care and tumor staging by medical institutions, and signaling that the implementation of guidelines should continue to increase and be targeted.

Foreign studies have not yet focused on the completeness of pre-treatment clinical TNM staging and pathology type, but some studies have analyzed the appropriateness of biomarker testing among patients with advanced stage cancer; only 20.9% of patients with advanced disease in this study underwent biomarker testing, much lower than the 68.7% in the United States during the same period. This may be due to the high cost of such tests and the fact that they are not covered by medical insurance in China, resulting in their rejection by most patients (42). It is worth noting that the consistency of the diagnosis and guidelines experienced by patients in the oncology specialist hospital was very high, which may be because specialist hospitals are more focused on the professional and characteristic diagnosis and treatment of tumors, the discipline is developing rapidly, and the technology and standardization of diagnosis and treatment are higher (43, 44).

Other studies in the United States have reported that approximately 44.7%–76% of patients underwent GCT; the degree of GCT acceptance varies by cancer type and stage, and this determines the choice of appropriate treatment (23, 45–47). Our study is concordant with previous findings that the likelihood of undergoing GCT decreases with disease progression in NSCLC cases, was higher in stage I–II NSCLC than in stage III–IV NSCLC, this may be related to more direct patient selection and increased expectations of treatment outcomes. The majority of patients who did not undergo GCT presented with advanced disease, with quality of life (QOL) rather than survival likely to be an important consideration, and a previous study reported that patient preference for treatment was the most common reason for not seeking expert advice (48).

Sublobectomy was the most common non-GCT in early stage patients, accounting for 96.6% of stage I non-GCT and 13.1% of stage I surgery patients, an improvement over a previous study in Shanghai (49). However, in recent years there have also been studies showing that sublobar resection is noninferior to lobectomy in terms of disease-free survival (risk ratio for disease recurrence or death, 1.01; 90% [CI], 0.83–1.24), and although these results have not yet been reflected in the guidelines, this trend has been observed and may be influencing the choice of resection (50). In addition, non-GCT for advanced lung cancer is primarily non-targeted, due in large part to the lack of definitive pathologic molecular testing.

In our study, the proportions of patients who underwent both GCD and GCT, GCD alone, GCT alone and neither GCD nor GCT were 36.7%, 11.4%, 33.5% and 18.4%, respectively. A study by Meadows-Taylor et al. showed that these rates for NSCLC in Tennessee from 2014 to 2019 were 61.2%, 12.7%, 17.2% and 8.9%, respectively (51). This indicates that the overall procedures in the first course of diagnosis and treatment of lung cancer in China are poorly adherent to the guidelines and require enhanced supervision and management.

Our follow-up results suggest that either GCD or GCT reduces the risk of death by more than a quarter, concordant with previous studies (24, 52). However, subgroup analysis showed that the effect of GCD on survival was not significant, probably because we focused on GCD as the completeness of the pre-treatment diagnostic report, a procedure that is less likely to affect patient prognosis directly and more likely to affect patient survival indirectly by influencing treatment choice. For advanced NSCLC, highly effective targeted therapies have been approved in many places for patients with EGFR-activating mutations, ALK fusions and ROS1 fusions (53, 54). The development of novel targeted therapies for patients with advanced NSCLC with specific genetic alterations (driver mutations) has greatly improved response rates and survival compared to previously available treatments (6, 55). However, the accessibility of targeted therapies for lung cancer depends on the accurate identification of patient biomarkers through molecular testing (56). Very few patients with advanced NSCLC in this study underwent biomarker testing, with negative impact on their survival. For stage I NSCLC, those who received both GCD and GCT showed the lowest risk of death, suggesting that GCD and GCT were complementary in their association with improved survival in stage I. Therefore, providing both GCD and GCT to patients with stage I NSCLC may have higher benefits for them, especially with the development of lung cancer screening programs in recent years, increasing numbers of cases are being detected at an early stage, with 63.7% of patients in this study being in stage I (57). Although the results of this study show that GCT has a greater survival benefit compared to GCD in almost all stages of NSCLC, as mentioned earlier about the positive impact of molecular pathology testing on survival, proper staging and pathology testing prior to treatment is also important for the survival of NSCLC patients (51).

Therefore, it is critical to provide NSCLC patients with the most appropriate diagnostic procedures and deploy optimal treatment within a reasonable limit of the specific clinical situation, patient preferences and available resources. In the future, more rigorous, prospectively designed and executed studies at the intersection of these two critical components of lung cancer care are needed to validate the impact of GCD and GCT on patients’ survival.

There are some limitations to this study. First, owing to the lack of large databases such as SEER and NCDB in China, our cohort used data from only one province, Liaoning, with a small sample size. As such, there is some uncertainty about comparability when comparing with previous results based on large databases, but we believe that the exploration of the application of clinical practice guidelines in China is complementary to this area of research. The patients’ performance status and comorbidities were used as part of the rationale for treatment, which may be related to a patient’s decision to avoid aggressive treatment, but this information is not available in our data source. In addition, we excluded patients who did not undergo any treatment, although “no treatment” may be considered appropriate treatment given the heterogeneity of patients, therefore, we may have overestimated the proportion of patients undergoing GCT. Future research could collect more comprehensive information, including individual patient income, education, functional status, treatment preferences, and factors related to the doctor, such as the degree of expertise, treatment choice, etc. Besides that, the cases in this study are from 2017-2018, and the use of immune checkpoint inhibitors related to lung cancer is not as widespread; however, with their increasing use as first-line therapies in lung cancer in recent years, the results of this study may change, relevant and more recent studies are still needed for the future.

## 5 Conclusion

This study is the first in China to assess concordance with guidelines for the first course of lung cancer diagnosis and treatment. We found a poor level of guideline concordance for NSCLC diagnosis and treatment in China, which was lower than that in the United States during the same period. Patients with early-stage NSCLC, as well as those from oncology specialist hospitals, are more likely to undergo GCD and GCT. Undergoing GCD and GCT improves the survival of patients with NSCLC. Therefore, there is a need to establish an oncology diagnosis and treatment data management platform in China to monitor, evaluate and promote the use of clinical practice guidelines in healthcare institutions in order to maximize the survival rate of lung cancer patients in China.

## Data availability statement

The original contributions presented in the study are included in the article/supplementary material. Further inquiries can be directed to the corresponding author.

## Ethics statement

The studies involving humans were approved by the Ethics Committee of Liaoning Center for Disease Control and Prevention. The studies were conducted in accordance with the local legislation and institutional requirements. The participants provided their written informed consent to participate in this study.

## Author contributions

HM: Conceptualization, Formal analysis, Investigation, Methodology, Visualization, Writing – original draft. XY: Conceptualization, Formal analysis, Investigation, Methodology, Visualization, Writing – original draft. YL: Data curation, Formal analysis, Investigation, Methodology, Visualization, Writing – original draft. BZ: Data curation, Formal analysis, Investigation, Methodology, Visualization, Writing – original draft. LL: Data curation, Formal analysis, Investigation, Methodology, Visualization, Writing – original draft. MZ: Data curation, Formal analysis, Investigation, Writing – original draft. QW: Data curation, Formal analysis, Investigation, Writing – original draft. QC: Data curation, Formal analysis, Investigation, Writing – original draft. LY: Conceptualization, Funding acquisition, Methodology, Supervision, Writing – review & editing. WS: Conceptualization, Methodology, Project administration, Writing – review & editing. GP: Conceptualization, Funding acquisition, Methodology, Supervision, Writing – review & editing.
